# Draft genome sequence of *Streptomyces carpaticus* FH1

**DOI:** 10.1128/mra.01054-24

**Published:** 2024-12-31

**Authors:** Farwa Hassan, Hazrat Ali, Noor Hassan, Anam Saqib, Muhammad Hamid Rashid, Aniqa Nawaz, Rubab Zahra Naqvi

**Affiliations:** 1 Industrial Biotechnology Division, National Institute for Biotechnology and Genetic Engineering-College, Pakistan Institute of Engineering and Applied Sciences, Islamabad, Pakistan; 2 Agriculture Biotechnology Division, National Institute for Biotechnology and Genetic Engineering-College, Pakistan Institute of Engineering and Applied Sciences, Islamabad, Pakistan; DOE Joint Genome Institute, Berkeley, California, Berkeley, California, USA

**Keywords:** phytopathogens, *Streptomyces*, antimicrobial agents, kanamycin

## Abstract

Phytopathogens with multi-drug resistance are emerging frequently, resulting in various disease outbreaks. Hence, exploring new antimicrobials is urgent. Here, we present the draft genome sequence of *Streptomyces carpaticus* FH1 strain, with the potential to produce various antimicrobial compounds. Genomic annotation revealed various genes encoding kanamycin, ectoine, and naringerin.

## ANNOUNCEMENT

The water- and soil-borne phytopathogens and their subsequent infection have caused 20%–40% annual crop yield loss (1) around the world. Traditionally, different chemical pesticides have been used for plant protection, but there is a dire need to adapt biological means to overcome human and environmental health hazards (2). In this regard, bacteria in the genus *Streptomyce*s have been studied extensively as promising biocontrol agents based on their potential to produce a broad range of natural compounds (3). Here, we isolated the bacterial strain FH1 from wheat rhizospheric soil in a field area located within the premises of National Institute for Biotechnology and Genetic Engineering (NIBGE), Faisalabad, (31.3980 N 73.0257 E) Pakistan, by the end of March 2023 through serial dilution on Luria Bertani Agar (LBA) at 28°C ± 2°C followed by 24 h of incubation. Typical gray colonies, with a filamentous arrangement of cells and spores with oval or globular shapes, were observed by microscopy for strain FH1. Hence, the isolated strain was predicted to be a member of the *Streptomyces* genus prior to genome sequencing. The whole genome sequencing was performed to shed light on the molecular background of these traits as well as potential biosynthetic clusters involved in secondary metabolism.

The genomic DNA (gDNA) was extracted from a single colony of 24-h-old culture grown at 28°C in LBA plates, by using the GeneJET Genomic DNA purification kit (Thermo Scientific, Germany). The gDNA was not sheared, sequencing libraries were generated using the Nextera XT library preparation kit with a random selection approach, by following user guidelines, and sequencing was performed through Illumina Miseq instrument with paired-end (2 × 250 bp) configuration. The final genome coverage was 30×, and the estimated completeness of this genome was 100%. The quality of Illumina reads (422908 raw read pairs with 31.83 sequencing depth) was initially assessed by using FastQC v0.11.5 tool (4). The low-quality reads and adaptor sequences were removed through the Trimmomatic v0.36 tool (5) in the Galaxy database, and trimmed read pairs (399907) were used for assembly by using SPAdes (6), followed by the exclusion of <200 bp contigs; the resulting assembly quality was checked through QUAST tool (7). All the tools were run with default configuration. The assembly results revealed 74 contigs with 5.7 Mb genome size. The draft genome contains 5733977 nucleotides with a G + C content of 72.5 (Table 1). In addition, the genome annotation was performed by Prokaryotic Genome Annotation Pipeline (PGAP) pipeline (8), revealing 5,261 total genes and 5,070 protein-coding sequences, 5 rRNA operons, and 57 tRNA loci.

**TABLE 1:** Genomic features of *Streptomyces carpaticus* FH1 strain predicted by the PGAP pipeline

Genome	Features
Number of contigs	74
Genome size (Mb)	5.7 Mb
N_50_	196 kb
GC (%)	72.5
tRNA	57
rRNA	5
CDS	5,189
Genome coverage	31.83×
Genome completeness	100%

We searched the genome sequence of the *Streptomyces carpaticus* FH1 strain by using the bioinformatics tool antiSMASH v6.0.0 (bacterial version) (9). The analysis predicted 20 different kinds of Biosynthetic Gene Clusters (BGCs). Collectively, these clusters are predicted to be involved in *de novo* synthesis of kanamycin, coelibactin, ohmyungsamycin A and B, deoxyhangtamycin, chlortetracycline, ectoine, puromycin, butyrolactol A, valinomycin, naringerin, hopene, ikarugamycin, geldanamycin, legonoxamine A, desferrioxamine B, egonoxamine B, and many more.

The *S. carpaticus* FH1 genome sequence uncovered new avenues to explore bioactive compounds produced by it. Furthermore, it will allow us to investigate the molecular basis to understand their biosynthesis, functionality, and transportation to exploit the FH1 strain, as a potential biocontrol candidate to cope with infections produced by phytopathogens.

## Data Availability

The complete sequence report of Streptomyces carpaticus FH1 can be found at NCBI/GenBank under the accession number JBHGBT000000000 (first version), BioProject number PRJNA1160071 and BioSample number SAMN43615635, Sequence Read Archive (SRA) for raw sequence reads of Streptomyces carpaticus FH1 SRX27019370 and BioProject number PRJNA1196100.
